# Eplerenone Attenuates Hepatic Oxidative Stress and Mitochondrial Dysfunction Following Renal Ischemia-Reperfusion Injury in Rats

**DOI:** 10.34172/apb.025.45926

**Published:** 2025-12-21

**Authors:** Alireza Barati, Seyed Mohammadmahdi Meybodi, Kimia Moradi, Sana Nouraei, Mohammad Bakhshi, Soheila Montazersaheb, Sepideh Zununi Vahed

**Affiliations:** ^1^Faculty of Veterinary Medicine, Tabriz Branch, Islamic Azad University, Tabriz, Iran; ^2^Molecular Medicine Research Center, Tabriz University of Medical Sciences, Tabriz, Iran; ^3^Kidney Research Center, Tabriz University of Medical Sciences, Tabriz, Iran

**Keywords:** Aldosterone antagonist, Liver, Distant organ, Mitochondrial dysfunction, Sirtuins, PGC-1α, Opa-1

## Abstract

**Purpose:**

Acute kidney injury (AKI) is frequently complicated by systemic manifestations, including hepatic dysfunction, largely due to ischemia-reperfusion (I/R) injury. This study investigated the hepatoprotective effects of eplerenone, a selective aldosterone receptor antagonist, in a rat model of renal I/R.

**Methods:**

Male Wistar rats (n=24) were randomly divided into four groups: sham, I/R, eplerenone+I/R, and eplerenone alone. A single intraperitoneal dose of eplerenone (100 mg/kg) was administered one hour before ischemia induction. Hepatic injury was evaluated by histopathology, liver function enzymes (ALT, AST, ALP), oxidative stress parameters, sirtuins, and key mitochondrial and apoptotic signaling proteins.

**Results:**

Renal I/R resulted in significant hepatic injury, characterized by histological alterations, increased liver enzymes, oxidative stress, and activation of inflammatory and apoptotic pathways. Pretreatment with eplerenone markedly improved hepatic outcomes by lowering ALT, AST, and ALP levels, preserving liver morphology, suppressing NF-κB and caspase-3 expression, and enhancing antioxidant and mitochondrial protective mechanisms, particularly through Nrf2 and Sirt-3/PGC-1α/Opa-1 pathways. The results indicated that eplerenone administration significantly improved liver function and histology following kidney I/R injury. Caspase-3 and NF-κB levels were downregulated following eplerenone administration. It also elevated the antioxidant capacity and protein levels of Nrf-2, HSP-70 (*P*<0.001), mitochondrial biogenesis factor (PGC-1α), sirtuin-1 (*P*<0.05), and dynamics factor (Opa-1, *P*<0.001), while decreasing Drp-1 levels (*P*<0.05) in the liver after kidney I/R injury.

**Conclusion:**

Eplerenone demonstrated strong hepatoprotective effects against renal I/R-induced liver injury. Its antioxidant, anti-inflammatory, and mitochondria-stabilizing actions highlight its potential as a therapeutic agent for preventing hepatic dysfunction associated with AKI and underline the importance of targeting distant organ injury in acute renal pathophysiology.

## Introduction

 Acute kidney injury (AKI) is a major pathophysiological event in many clinical situations, with ischemia-reperfusion (I/R) injury being its leading cause. I/R injury is triggered by surgery, transplantation, and other pathological conditions.^1^ AKI is not an isolated problem but contributes to a series of events and abnormalities in the electrolyte and acid-base equilibrium, leading to death. The reason behind AKI-related mortality is not impaired renal function or treatment complications but rather an imbalance in the immunologic, humoral, and hemodynamic systems. I/R injury also contributes to endothelial injury, organ cell death, excessive generation of ROS, immune system dysregulation, increased leukocyte infiltration rate, and activation of pro-inflammatory and pro-apoptotic cascades in distant organs.^2^

 Several studies have revealed crosstalk between the liver and kidneys through the regulation of body metabolism, homeostasis, and excretion of toxic products and drugs.^3^ The combination of I/R injury and liver disease is likely associated with more serious clinical problems and mortality.^4^

 Mitochondria are essential organelles involved in ATP production and the preservation of the hemostatic state in the body. It has been well documented that dysfunction of the mitochondria is a prominent event in many diseases.^5,6^ Sirtuins (Sirts) with different subcellular localizations are involved in many cellular processes through post-translational modifications. Sirts promote the transcription of genes involved in the regulation of mitochondrial biogenesis. Sirt-1 and Sirt-3 are known to have regulatory roles in mitochondrial function and inflammatory responses.^7^ These modulators also contribute to hepatic lipid pathways; therefore, their absence may adversely affect liver function.^8^ As dynamic organelles, the mitochondria frequently divide and fuse together. Activated fission stimulates mitochondrial dysfunction, while fusion is a protective mechanism that reduces mitochondrial fission.^9^ Opa-1 facilitates fusion, and Drp-1 is a crucial regulator of fission of mitochondria; therefore, stabilizing Opa-1 might protect liver tissues from stress-induced damage.^10^ It has been reported that reduced levels of PGC-1α (a master regulator of mitochondrial biogenesis) are associated with overexpression of Drp-1 and mitochondrial fission^11^, contributing to hepatic fibrosis.^12^

 Eplerenone is a highly selective antagonist of the aldosterone receptor, chemically derived from spironolactone.^13,14^ Aldosterone contributes to hypertension and organ damage via sodium reabsorption, endothelial dysfunction, and vascular remodeling, while eplerenone antagonizes these effects by competitively binding to mineralocorticoid receptors (MRs).^15^ The MR pathway plays a significant role in mediating multi-organ damage following AKI, including injury to distant organs such as the liver.^16^ Eplerenone is a selective MR antagonist known to counteract key pathological processes—oxidative stress, inflammation, and fibrosis—that drive organ injury in I/R scenarios. Preclinical studies in various liver injury models, including toxin-induced hepatotoxicity (e.g., thioacetamide) and cirrhosis or hypoxia-induced injury, highlight the hepatoprotective potential of eplerenone.^17,18^ Moreover, eplerenone’s clinical use in chronic conditions linked to oxidative and inflammatory injury^19^ supports its safety profile and suggests potential applicability in acute injury settings. Given the expression of MR in liver tissue and its pivotal role in multi-organ injury after AKI, these preclinical and clinical examples strongly support the choice of eplerenone to target MR signaling and mitigate liver damage following AKI specifically.

 Considering the complicated relationship between the kidney and liver as distant but metabolically interconnected organs, a deeper understanding of this crosstalk may provide new opportunities for improved therapeutic strategies in AKI. Accordingly, it is essential to develop clinically practical approaches that can mitigate the systemic consequences of renal injury on remote organs. In this context, the present study aimed to evaluate the hepatoprotective effects of eplerenone against renal I/R injury in rats, with a particular focus on mitochondrial-related mechanisms in liver tissue, acknowledging the vital role of the liver in drug metabolism and detoxification.

## Methods

###  Experimental animals and induction of ischemia-reperfusion

 Male Wistar rats (3–4 months old, 230 ± 20 g, n = 24) were obtained from the Pasteur Institute (Tehran, Iran). Animals were acclimatized for 7 days under controlled conditions (21 ± 1 °C, 50% humidity, and a 12/12 h light/dark cycle) in accordance with the Animal Care and Use guidelines. Following adaptation, the rats were randomly allocated into four experimental groups: (1) sham, (2) I/R, (3) eplerenone (Normon, Madrid, Spain) + I/R, and (4) eplerenone. According to previous studies, 100 mg/kg of eplerenone (single dose) was administered intraperitoneally 1 hour before I/R induction.^20-24^ The animals’ body temperature was carefully controlled during all surgical operations, the 45-minute ischemia induction, and the 6-hour renal blood flow reperfusion. Each animal was anesthetized with xylazine (Kela, Belgium) and ketamine (Bremer Pharma, Germany) at 10 and 90 mg/kg, respectively. In the sham group, 1 h before kidney vascular manipulation (abdominal surgery with kidney displacement but no ischemia) without any clamps, we injected normal saline (0.9 %) with the same volume of eplerenone administration (0.4 ml through IP) to simulate injection stress. In the I/R groups (groups 2 and 3), the abdominal wall was cut, and only the entire left renal pedicle (vein and artery) was blocked for 45 min using forceps (nontraumatic) clamps (unilateral renal ischemia/reperfusion). Ischemia was successfully induced when the red color of the kidneys turned pale. After removing the clamps, renal blood flow reperfusion was re-established for 6 h. The eplerenone group (group 4) received only a dose of eplerenone (100 mg/kg, IP) without any surgical operations for examination of drug effects exclusively on the liver. For biochemical analysis, blood samples were collected at the end of 6 h of renal reperfusion. The rats were euthanized, and their livers were collected and stored for further studies. All procedures were performed as described by Barati et al.^20^

###  Histopathological study

 The impact of eplerenone on histopathological alterations in the liver was evaluated by H&E staining. Histopathological changes were assessed using an Olympus light microscope (Tokyo, Japan). Semi-quantitative scoring was adapted from the Suzuki criteria for hepatic I/R injury^25^, where congestion of blood sinusoidal congestion (0–4), cytoplasmic vacuolization (0–4), and hepatocyte necrosis parameters are graded 0 to 4. The defined ranges indicate; 0 = No damage; 1 = Minimal congestion, minimal vacuolization, and single cell necrosis; 2 = Mild congestion, mild vacuolization, less than 30% necrosis; 3 = Moderate congestion, moderate vacuolization, less than 60% necrosis; 4 = Severe congestion, severe vacuolization, more than 60% necrosis. The investigators who evaluated tissue samples and performed biochemical assays and data analyses were blinded to the treatment group assignments of the samples.

###  Liver functional assays 

 To examine liver function, liver enzyme levels, including ALP (alkaline phosphatase), ALT (alanine transaminase), and AST (aspartate aminotransferase), were examined in serum samples of all experimental groups using a photometric method and commercial kits (Pars Azmoon, Iran).

###  Oxidative stress examination

 Superoxide dismutase (SOD, ZellBio GmbH, Germany, ZB-SOD-96A), catalase (CAT, Cusabio CSB-E13439r), glutathione peroxidase (GPX, ZellBio GmbH, Germany, ZB-GPX-A48), and total antioxidant capacity (TAC, ABTS method, E-BC-K219) were assessed using the colorimetric assays. Furthermore, liver tissue levels of malondialdehyde (MDA) were evaluated by commercial kits (ZellBio GmbH, Germany, D-89075). All processes were performed based on the previously published studies by this team.^26,27^

###  Western blot analysis for the determination of different proteins 

 The impact of eplerenone on protein levels of Drp-1, HSP-70, NF-κB, Nrf-2, Opa1, PGC-1, Sirt-1, and Sirt-3 was evaluated in liver tissue using western blotting as previously described.^28,29^ Briefly, liver tissues were lysed using RIPA buffer containing a protease inhibitor (1% cocktail), and then the total cellular protein was extracted. Lysates were centrifuged for 10 min (at 12000 rpm), and the concentration of proteins in the supernatant was measured with the BCA protein assay (Rockford). Subsequently, each protein sample (12 mg/ml) was subjected to SDS-PAGE (10%) and then transferred to polyvinylidene fluoride membranes (USA). To inhibit nonspecific protein interactions in the membrane, skim milk was applied to block the membranes. Next, the membrane was incubated overnight with primary antibodies against β-actin (sc-47778), Drp-1 (sc-271583), HSP-70 (sc-66048), NF-κB (sc-8008), Nrf-2 (sc-365949), Opa-1 (sc-393296), PGC-1α (ab54481), Sirt-1 (sc-74465), and Sirt-3 (sc-365175). After washing ( × 3) with Tris-buffered saline-Tween 20 (TBST), the primary antibodies were detected by staining with a secondary antibody [anti-rabbit IgG-HRP (sc-2357)] at room temperature at a 1:1,000 dilution for two hours. Subsequently, the protein bands were visualized with an X-ray film (Roche, UK), following membrane washing. Western blot analyses in this study were quantitatively assessed using densitometry software to measure the intensity of specific protein bands relative to the appropriate loading control (β-actin). Band intensities were corrected for background and normalized to the loading control to ensure accurate comparison between groups. These normalized intensity values were then expressed as fold changes relative to the sham or control group. Replicates were included to ensure data reliability and reproducibility.

###  Statistical analysis

 All values are represented as the mean ± standard deviation. Statistical analysis was performed using one-way analysis of variance (ANOVA) followed by Tukey’s post-hoc multiple comparison assay. Kruskal-Wallis nonparametric test was used to detect significant changes of Suzuki score. IBM SPSS (version 16.0; SPSS, Inc.) was used for statistical analysis, with a significance level set at *P* < 0.05. All procedures were repeated three times to ensure accuracy and reproducibility.

## Results

###  Eplerenone administration diminishes kidney I/R-induced histological damage in the liver

**Figure 1:**
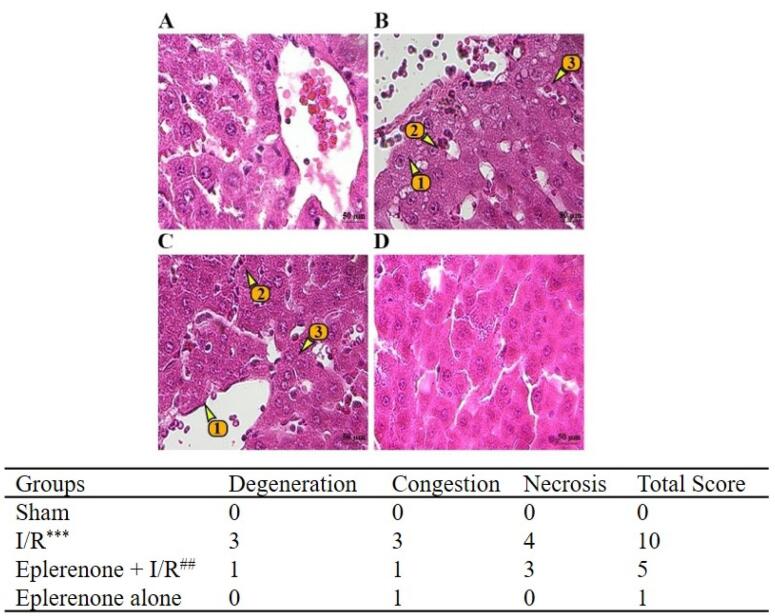
Histopathological study of eplerenone treatment on kidney I/R-induced injury in the liver. H & E staining of liver sections of all experimental animals is depicted as follows: (A) sham, (B) I/R, (C) Epl + I/R, and (D) Epl groups. The following parameters were shown on the liver tissues: 1. hepatocellular degeneration, 2. sinusoidal congestion, and 3. parenchymal necrosis. The table shows the histopathological scoring of liver injury (Suzuki method). Absence of parenchymal necrosis, sinusoidal congestion, or cytoplasmic vacuolization was given a score of 0, while severe sinusoidal congestion, severe cytoplasmic vacuolization, and more than 60% parenchymal necrosis received a score of 4. Each H&E-stained sample was assigned a total score by summing these three parameters, resulting in a cumulative range from 0 to 12. Data are presented as Median. ***P< 0.001 compared with the sham group, and ^##^*P*< 0.01 compared with the I/R induced animals (2). Epl, eplerenone; I/R: ischemia-reperfusion; H&E: hematoxylin and eosin (scale bar = 25µm × 400)

 The liver histological images of all experimental rats are shown in Figure 1A and 1B. In the I/R group, severe injury with moderate degeneration, severe congestion, and severe necrosis (total score of 11) were seen compared to the sham group (*P* < 0.001). Mild to moderate injury with minimal degeneration, mild congestion, and moderate necrosis (total score of 5) was seen in the Eplerenone + I/R group compared with those in the I/R group (*P* < 0.01). Mild injury with minimal congestion and no necrosis (total score of 1) was observed in the Eplerenone group. These findings imply that eplerenone treatment can ameliorate the abnormal hepatic morphology in I/R-induced rats.

###  The impact of eplerenone on liver function

**Figure 2:**
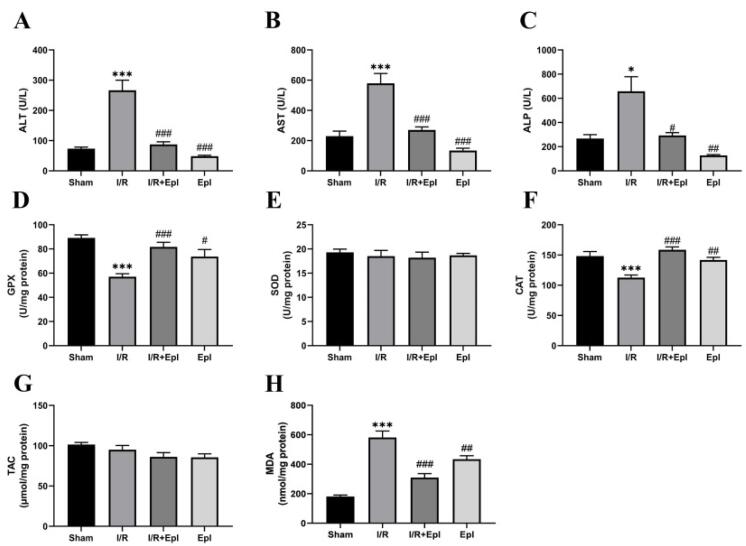
The effect of eplerenone on liver function and antioxidant status. The levels of (A) ALT, (B) AST, (C) ALP, (D) GPX, (E) SOD, (F) CAT, (G) TAC, and (H) MDA were examined in all studied groups. Mean ± SD are presented. *,^#^*P*< 0.05, **,^##^*P*< 0.01, and ***,^###^*P*< 0.001, *comparing with sham groups, ^#^ comparing with I/R induced rats. Epl: eplerenone; ALP: alkaline phosphatase, ALT: alanine transaminase, AST: aspartate aminotransferase, CAT: catalase, GSH: glutathione, GPx: glutathione peroxidase, I/R: ischemia-reperfusion, MDA: malondialdehyde, SOD: superoxide dismutase, TAC: total antioxidant capacity

 The results revealed that the levels of ALP, AST, and ALT in the I/R-induced rats were higher than those in the sham group (*P* < 0.05). In comparison with the I/R group, the eplerenone + I/R group exhibited a significant reduction in the levels of ALT (*P* < 0.001), AST (*P* < 0.001), and ALP (*P* < 0.05). Notably, in animals receiving eplerenone alone, there was a significant decline in the levels of ALT, AST, and ALP compared to the I/R group (*P* ≤ 0.01). Decreased levels of liver enzymes were also seen in the eplerenone group compared to the sham group; however, these were not statistically significant (Figure 2A-C).

###  Eplerenone improved oxidative stress in the liver after acute kidney injury

 Kidney I/R damage reduced the enzymatic activities of GPX and CAT significantly in comparison with those in the sham group (*P* < 0.001); however, eplerenone administration led to a substantial increase in their activities (*P* < 0.001). Notably, in animals receiving eplerenone alone in comparison to the I/R group, a significant increase was observed for GPX and CAT activities (*P* < 0.05 and *P* < 0.01, respectively). The SOD and TAC activities were not affected by eplerenone treatment (Figure 2; D-G). Renal I/R injury led to a significant increase in MDA levels in the liver of the I/R group compared with the sham group (*P* < 0.001). In contrast, eplerenone administration significantly decreased MDA levels in I/R-induced rats (*P* < 0.001). A significant reduction in MDA level was observed in rats that received eplerenone alone compared to the I/R (*P* < 0.01). However, increased levels of MDA were seen in rats who received just eplerenone compared to the rats in the sham group (*P* < 0.05), Figure 2H.

###  Eplerenone reduced apoptotic and inflammatory proteins in the liver after renal I/R-induced injury

**Figure 3:**
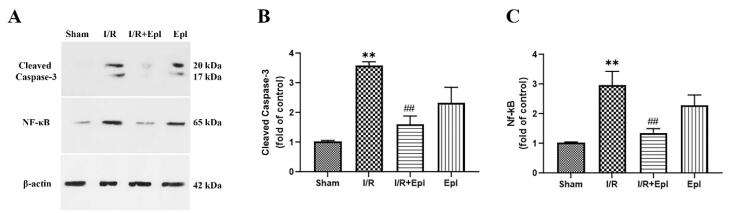
Evaluation of caspase-3 and NF-κB signaling proteins in rats subjected to eplerenone treatment. Representative western blotting is shown in (A). The expression level of cleaved caspase-3 (B) and NF-κB (C) were detected by western blot-assisted analysis. Mean ± SD are presented. *,^#^*P*< 0.05, **,^##^*P*< 0.01, and ***,^###^*P*< 0.001, *comparing with sham groups, ^#^ comparing with I/R induced rats. NF-κB: nuclear factor kappa-B, Epl, eplerenone; I/R: ischemia-reperfusion

 The caspase-3 and NF-κB levels were upregulated significantly in the I/R group compared with the sham group (*P* < 0.01). Following the administration of eplerenone, the protein levels of cleaved caspase-3 and NF-κB significantly declined (*P* < 0.01) compared to those in the I/R rats. No significant reduction in these proteins was observed in rats that received eplerenone alone compared to the I/R group. However, increased levels of caspase-3 and NF-κB were observed in the eplerenone group compared to the sham group (Figure 3).

###  Eplerenone improved mitochondrial-related proteins and Sirtuins in the liver after renal I/R-induced injury

**Figure 4:**
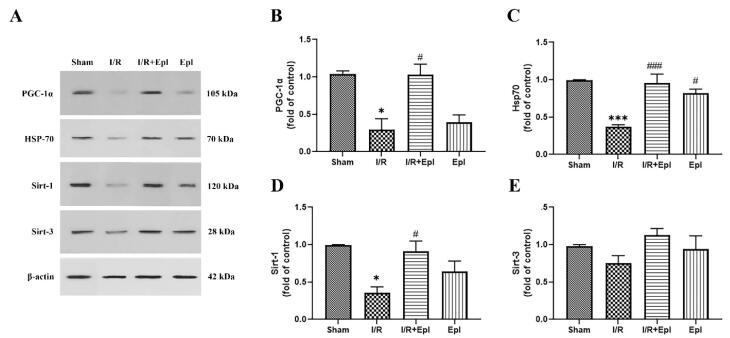
Impact of eplerenone on the liver heat-shock, PGC1- α. and sirtuin proteins. Schematic representation of the western (A) and their relative densities of PGC1- α (B) HSP70 (C), Sirt-1 (D), and Sirt-3 (E) in the studied groups. Mean ± SD are presented. *,^#^*P*< 0.05, **,^##^*P*< 0.01, and ***,^###^*P*< 0.001, *comparing with sham group, ^#^ comparing with I/R induced rats. Epl, eplerenone; I/R: ischemia-reperfusion. PGC-1α: peroxisome proliferator-activated receptor-gamma coactivator-1α, Opa-1: dynamin-like GTPase protein Optic Atrophy 1, Drp-1: dynamin-related/-like protein 1, HSP70: heat-shock protein 70, Sirt: sirtuin

 To determine the effect of eplerenone on sirtuins and mitochondria-related proteins, whose abnormal levels contribute to mitochondrial dysfunction, Western blot analysis was performed. Figure 4 shows that the levels of PGC-1α (B), HSP70 (C), Sirt-1 (D), and Sirt-3 (E) in the I/R-induced rats were lower than in the sham group (*P* < 0.05), while eplerenone could increase their expression at the protein level significantly (*P* < 0.05). No significant changes were detected in the protein expression of the abovementioned proteins in only eplerenone rats in comparison to the I/R group rats, except for the HSP70 protein (*P* < 0.05). However, decreased levels of PGC-1α and Sirt-1 were observed in the eplerenone group compared to the sham group.

**Figure 5:**
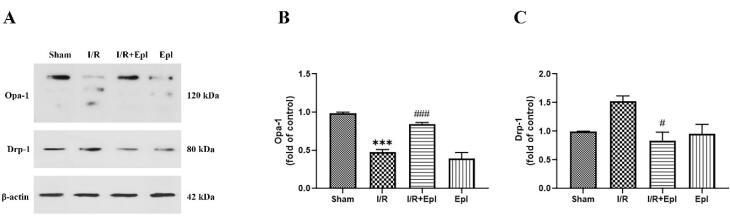
The impact of eplerenone on liver mitochondrial dynamic proteins. Schematic representation of the western blotting (A) and their relative densities of Opa-1 (B) and Drp-1 (C). Mean ± SD are presented. *,^#^*P*< 0.05, **,^##^*P*< 0.01, and ***,^###^*P*< 0.001, *comparing with sham groups, ^#^ comparing with I/R induced rats. Epl, eplerenone; I/R: ischemia-reperfusion

 The impact of eplerenone on mitochondrial dynamics is depicted in Figure 5 A-C. The level of Opa-1 (B) was significantly decreased in I/R-induced rats (*P* < 0.001) compared to that in the sham group, while an increase was detected in the level of Drp-1 (*P* > 0.05, Figure 5; B, C). Upon treatment with eplerenone, the protein level of Opa-1 was significantly increased (*P* < 0.001), while the level of Drp-1 was decreased (*P* < 0.05) compared to the I/R group. Eplerenone alone showed a decrease in the Opa-1 levels compared to the sham rats (*P* < 0.05).

###  Eplerenone-induced Nrf-2 activation in the liver after renal I/R-induced injury

**Figure 6:**
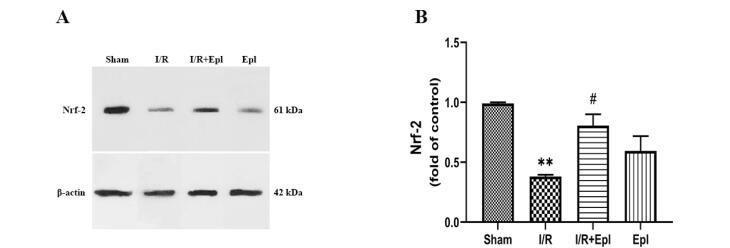
Evaluation of Nrf-2 signaling proteins in rats subjected to eplerenone treatment using western blotting analysis. Representative western blotting (A) and its relative densities (B) are shown. Mean ± SD are presented. *,^#^*P*< 0.05, **,^##^*P*< 0.01, and ***,^###^*P*< 0.001, *comparing with sham groups, ^#^ comparing with I/R induced rats. Epl, eplerenone; I/R: ischemia-reperfusion

 The impact of eplerenone administration on liver levels of Nrf-2 was also examined. The Nrf-2 level was dramatically decreased (*P* < 0.01) in I/R-operated rats compared with that in the sham group. In I/R rats treated with eplerenone, a significant elevation (*P* < 0.05) in the protein expression of Nrf-2 was seen (Figure 6). The eplerenone-treated rats showed no considerable alteration in levels of this protein compared to the sham and I/R groups.

## Discussion

 In the current study, eplerenone could protect the liver against kidney I/R-induced distant organ damage. Eplerenone could significantly represent anti-inflammatory, anti-oxidative (by affecting Sirt-1, Nrf-2, and downstream antioxidants), and anti-apoptotic impacts on I/R-induced liver damage. Moreover, eplerenone could significantly protect mitochondria against I/R-induced mitochondrial dysfunction by activating the Sirt-3/PGC-1α/Opa-1 signaling pathway in the liver.

 Eplerenone exerts its effect through competitive inhibition of aldosterone at the site of aldosterone receptors.^30^ In a CCl₄-induced liver injury model, pretreatment with eplerenone significantly reduced serum levels of liver enzymes and bilirubin, attenuated oxidative stress, restored glutathione, suppressed TNF-α, and improved histopathological damage.^17^ More recent studies have shown that eplerenone can reverse hypoxia-induced metabolic dysfunction in hepatocytes by restoring the expression of energy metabolism regulators, thereby partially normalizing glucose utilization, lactate production, and lipid accumulation under hypoxic stress.^18^

 The current study showed that the administration of eplerenone before renal I/R alleviated the histological evidence of liver injury and improved liver function. Similarly, in a study by Matono et al., eplerenone ameliorated hepatic fibrosis.^31^ The serum levels of ALT, ALP, and AST in rats treated with eplerenone were significantly lower than those in the I/R groups, indicating the benefits of eplerenone in improving I/R-induced liver function. These results are supported by a previously published study; however, the results are incongruent with those of other studies on hepatic enzymes.^32^ In addition, the results showed a significant decrease in all examined hepatic enzymes in the group that received the drug alone. This was attributed to the hepatoprotective effect of eplerenone, as reported previously. In contrast, Said et al. showed no significant alterations in liver enzymes following the administration of eplerenone in treated groups.^32^ It is plausible that the hepatoprotective effects attributed to eplerenone were, at least in part, secondary phenomena arising from attenuation of renal injury or systemic hemodynamic alterations mediated through RAAS (Renin-Angiotensin-Aldosterone System) inhibition.^33^ Therefore, delineating whether these effects reflect a direct hepatic action of eplerenone or indirect consequences of systemic modulation requires further mechanistic investigation.

 Oxidative stress and the higher production of ROS as a result of I/R injury have substantial roles in eliciting local or distant organ damage.^34,35^ Our findings showed reduced levels of GPX and CAT and elevated levels of MDA in I/R rats. However, eplerenone administration improved the antioxidant status in the liver tissues of eplerenone + I/R rats compared with that in the I/R group. These findings are in agreement with those of several studies that reported the antioxidant effects of eplerenone. Interestingly, while CAT and GPX were significantly altered, no changes were observed in SOD or TAC. This may be explained by the temporal regulation of antioxidant enzymes, as CAT and GPX are more responsive to acute hydrogen peroxide detoxification, whereas SOD and TAC may reflect longer-term adaptation.^36,37^ Mechanistically, these differential effects imply that eplerenone selectively enhances enzymatic defenses against peroxides rather than superoxide radicals, possibly reflecting targeted modulation of redox signaling and oxidative stress pathways.

 Apoptosis relies on caspase activity, which is the central mediator of renal I/R injury. It has also been shown that ROS production activates apoptotic genes and the caspase cascade.^38^ In this study, caspase-3 was overexpressed in rats with I/R; however, a dramatic decrease was observed in its expression in rats treated with eplerenone compared to the I/R group. These results are consistent with those reported by Ramírez et al., who reported the anti-apoptotic effect of eplerenone.^39^

 Mitochondria contribute significantly to cellular energy production and homeostasis. ATP depletion occurs during ischemia and a lack of oxygen, resulting in anaerobic respiration. Mitochondrial function is impaired following reperfusion, leading to excessive ROS production, induction of mitochondrial permeability transition pores, and cell death.^40^ Sirt-1 is a vital metabolic and cellular sensor that participates in the regulation of cellular functions and energy homeostasis. In this context, Khader et al. demonstrated that stimulation of the Sirt-1/ PGC-1α pathway could increase mitochondrial biogenesis, decrease energy deficits, and enhance energy metabolism, thereby attenuating I/R-induced renal injuries.^41^ PGC-1α, as a crucial metabolic mediator, is regulated by Sirt-1. Sirt-1 interacts with PGC1-α and deacetylates it, resulting in the overexpression of mitochondria-related proteins during ATP generation. The results of this study revealed a significant decline in the levels of Sirt-1 and PGC-1α after I/R injury, while eplerenone treatment increased the levels of these modulators.^42^ Consistent with our findings, Chen et al. reported that a higher level of PGC-1α ameliorates oxidative damage during I/R injury.^43^

 The mitochondria-protective effects of Sirt-3 have been reported in several studies. In addition, inhibition of Sirt-3 was seen after renal I/R injury. Sirt-3 has a considerable impact on mitochondrial function and energy metabolism.^44^ It has been reported that overexpression of Sirt-3 mitigated I/R-induced kidney damage through various events, including reduction of ROS, increased antioxidant capacity, sustained mitochondrial membrane integrity, and inhibition of I/R-triggered oxidative stress and mitochondrial-related apoptosis. Our results showed an increase in the level of Sirt-3, but this was not statistically significant. Furthermore, Sirt-3 can preserve the mitochondrial homeostatic status against I/R injury by enhancing the Opa-1-triggered fusion of mitochondria. Several studies have shown that mitochondrial fusion has a protective mechanism that reduces mitochondrial fission, as activated fission leads to mitochondrial dysfunction.^9^ In other words, the inhibition of Opa-1, the primary regulator of mitochondrial fusion, represses the mitochondria-protective effects of Sirt3, resulting in mitochondrial dysfunction.^45^ Our findings support this, in which Opa-1 expression was downregulated in I/R injury and increased in the eplerenone + I/R group. We found that levels of Drp-1, a crucial mitochondrial fission factor, decreased following eplerenone administration. This is another mechanism that accounts for the decrease in fission, observed in I/R, that is consistent with previous findings.^10^ Interestingly, no alterations were observed in those taking eplerenone alone, suggesting that other events may be involved in the reduction of Drp-1.

 ROS-induced oxidative stress causes tissue damage by activating the NF-κB signaling pathway. Relying on these factors and considering the contributory role of NF-κB in apoptosis regulation, we determined NF-κB expression. We found that the administration of eplerenone decreased the expression of NF-κB in eplerenone + I/R rats. This supports the findings of a previous study.^46^ No alterations were observed in rats that received only eplerenone. Several studies have demonstrated that Nrf-2 activation ameliorates I/R injury by hindering the NF-κB pathway and I/R-induced apoptosis, indicating the beneficial effects of Nrf-2 in I/R-mediated damage.^47^ Our results were consistent, implicating the protective function of eplerenone.

 These molecular changes highlight key pathways involved in liver injury and the therapeutic effects of mineralocorticoid receptor antagonism, enhancing our understanding of the drug’s protective mechanisms. By inhibiting the RAAS pathway, eplerenone could reduce systemic inflammation and oxidative stress alterations that worsen liver injury, thereby exerting indirect protective effects on the liver secondary to improved systemic and renal parameters. At the same time, MRs are expressed in liver tissue; thus, eplerenone may have direct hepatoprotective effects by antagonizing aldosterone-mediated oxidative stress, inflammation, and fibrosis locally within the liver. Considering these points, the hepatic effects observed in this model may arise from a combination of both direct actions on liver cells and indirect systemic effects via RAAS inhibition and blood pressure modulation. Without direct measurement of blood pressure or systemic RAAS components in our study, we cannot fully separate these contributions, but existing literature supports the dual mechanism of action.

 In normal unstressed tissue, physiological MR signaling plays essential roles in maintaining cellular metabolism, mitochondrial function, and homeostasis. Administration of eplerenone in this context may transiently disrupt this balance by blocking basal aldosterone-MR activity, which is necessary for normal antioxidant defense and cellular signaling. This disruption can lead to a mild increase in oxidative stress markers (e.g., MDA), apoptotic enzymes (e.g., caspase-3), and pro-inflammatory factors (e.g., NF-κB), along with decreased expression of mitochondrial biogenesis and antioxidant regulators such as PGC-1a and sirtuin 1. Thus, eplerenone may induce mild oxidative, apoptotic, and inflammatory changes in the normal liver but exert protective effects when pathological stress pathways are active, explaining the differences seen between the sham and eplerenone groups in this study. These results may reflect disruption of basal mineralocorticoid receptor signaling, which plays a physiological role in maintaining redox balance and cellular homeostasis.^46^ This interpretation aligns with published evidence showing that MR antagonists, including eplerenone, can have variable effects depending on tissue state, stress exposure, and treatment regimens, highlighting the importance of context in evaluating drug responses. ^17,18,32,48,49^ Such context-dependent effects suggest that eplerenone may have differential impacts under physiological versus pathological conditions, and this aspect should be carefully considered when extrapolating the data.

 This study has several important limitations that warrant careful consideration. The relatively small number of animals used may reduce the statistical power of the findings, although the group size was consistent with prior renal and hepatic I/R models.^20,50^ The absence of a formal power analysis limits the robustness of the conclusions. Future work should employ larger cohorts with appropriate power calculations to provide stronger validation. Furthermore, liver-specific functional markers such as serum albumin and bilirubin, along with detailed histological scoring for fibrosis and steatosis, were not included. The lack of these assessments restricts the strength of the hepatoprotection claim and introduces a potential risk of overinterpretation. It is also important to note that the present experimental design was restricted to a 6-hour reperfusion, focusing primarily on early molecular and histological changes, rather than long-term hepatic function or chronic pathological alterations. Validation in chronic models that incorporate comprehensive functional markers and fibrosis/steatosis scoring will therefore be essential for a more complete evaluation of hepatoprotection.^31,41^

 Another significant limitation is the lack of systemic parameters such as blood pressure and circulating RAAS peptides since RAAS activation and hemodynamic alterations are well-recognized contributors to distant organ injury following AKI.^2,16^ It remains challenging to fully differentiate direct hepatic protection from indirect systemic effects of eplerenone. This limitation is particularly important because it directly affects mechanistic interpretation. Incorporating systemic hemodynamic monitoring and RAAS profiling in future investigations will be necessary to clarify these mechanisms and provide a more precise interpretation of the drug’s protective effects. Finally, the relatively high dose of eplerenone employed in this study (100 mg/kg), although validated in previous oxidative stress and I/R models^17,21^, may not directly correspond to clinically relevant dosing. The translational applicability of our results is therefore limited, and caution is required when interpreting the clinical implications. Future studies should explore dose–response relationships, apply more clinically feasible administration routes (e.g., oral gavage or intravenous delivery), and evaluate long-term safety and efficacy to define better the therapeutic potential of eplerenone in both experimental and clinical contexts.

## Conclusion

 The present study suggests that a single dose of eplerenone may exert hepatoprotective effects against renal I/R injury. The beneficial effect of eplerenone is likely mediated by activating Sirt-1, which activates PGC-1α. PGC-1α cooperates with Nrf2 to promote mitochondrial biogenesis and antioxidant defenses. Sirt-3 in mitochondria further enhances mitochondrial dynamics and oxidative stress resistance via Opa-1 activation and regulation of fission/fusion machinery (Drp-1). HSP-70 is upregulated to assist in maintaining protein homeostasis. This network collectively maintains mitochondrial and cellular health in kidney I/R-induced liver injury by balancing oxidative stress, inflammation, and cell survival. Further studies are needed to investigate the therapeutic potential of eplerenone after I/R injury in distant organs.

## Availability of Data and Materials

 The datasets used and/or analyzed during the current study are available from the corresponding author on reasonable request.

## Competing Interests

 The authors declare that they have no financial and non-financial conflicts of interest.

## Consent for Publication

 Not applicable.

## Ethical Approval

 The study protocol was applied by the ethical committee at Tabriz University of Medical Sciences, Tabriz, Iran (Ethic Code No: IR.TBZMED.AEC.1400.018). Every effort was devoted to minimizing the discomfort and pain caused to the animals. All procedures were strictly carried out in accordance with the ARRIVE reporting guidelines.
